# Gastric ischaemia as an unusual presentation of median arcuate ligament compression syndrome

**DOI:** 10.1259/bjrcr.20160005

**Published:** 2016-07-25

**Authors:** Cayetano Sempere Ortega, Ignacio Gallego Rivera, Mahmoud Shahin

**Affiliations:** ^1^Department of Radiology, Hospital Universitario Ramón y Cajal, Madrid, Spain; ^2^Department of Radiology, ERESA Grupo Médico, Valencia, Spain

## Abstract

Median arcuate ligament compression syndrome is an anatomical and clinical entity defined by a combination of extrinsic compression of the coeliac axis by the median arcuate ligament and clinical manifestations. The majority of patients with features of compression experience no symptoms. The most common clinical symptoms when present are epigastric pain, nausea, vomiting and weight loss. Hypertrophy of the median arcuate ligament is a rare cause of chronic abdominal pain. We present a case of an elderly male patient who presented with acute epigastric pain, and gastric and intrahepatic portal pneumatosis on CT imaging. Emphysematous gastritis, caustic ingestion and other causes of this imaging presentation were ruled out. Imaging also showed chronic compression of the coeliac axis with compensatory hypertrophy of the gastroduodenal artery. Gastric ischaemia is a rare presentation of this syndrome, which occurs owing to the failure of compensatory mechanisms and resultant ischaemic injury to a virtual watershed vascular territory of the gastric wall. Conservative management was performed, including volume restoration, intravenous proton pump inhibitor therapy, broad-spectrum antibiotic therapy and blood transfusion. No surgical or endovascular interventional procedures were carried out. The patient showed clinical improvement soon after the initiation of treatment. Disappearance of the imaging findings was documented 2 weeks after treatment. Complete endoscopic recovery and absence of clinical alterations were observed during follow-up after 3 months.

## Clinical presentation

A 73-year-old male patient presented to our hospital with complaints of severe acute epigastric pain after an episode of dizziness, sweating and syncope.

He reported no significant medical history. The patient had been well until 2 days before, when he began to experience an intense watery diarrhoea unaccompanied by fever or pathologic products and without other gastrointestinal symptoms. Early in the morning, after a bowel movement, he experienced an episode of dizziness and sweating followed by intense weakness, which made him unable to stand. He did not lose consciousness. Immediately after this episode, he began to suffer from progressive epigastric pain, which become severe in the next few hours. He did not report previous or recent melena, haematemesis or haematochezia. The physical examination demonstrated marked hypotension, orthostatism and paleness. Body temperature and white blood cell count were normal. Systolic–diastolic blood pressure levels were 120/70 mmHg in the supine position and 77/57 mmHg in the standing position. The pain was intense, located in the epigastric region and not radiating. No signs of peritoneal irritation were observed. The laboratory tests showed microcytic anaemia with a haemoglobin level of 9 g dl^–1^. Normal values of liver enzymes, lactate levels and amylase were observed. Lipase values were not measured. Blood cultures were not obtained.

## Investigations/imaging findings

**Figure 1.**
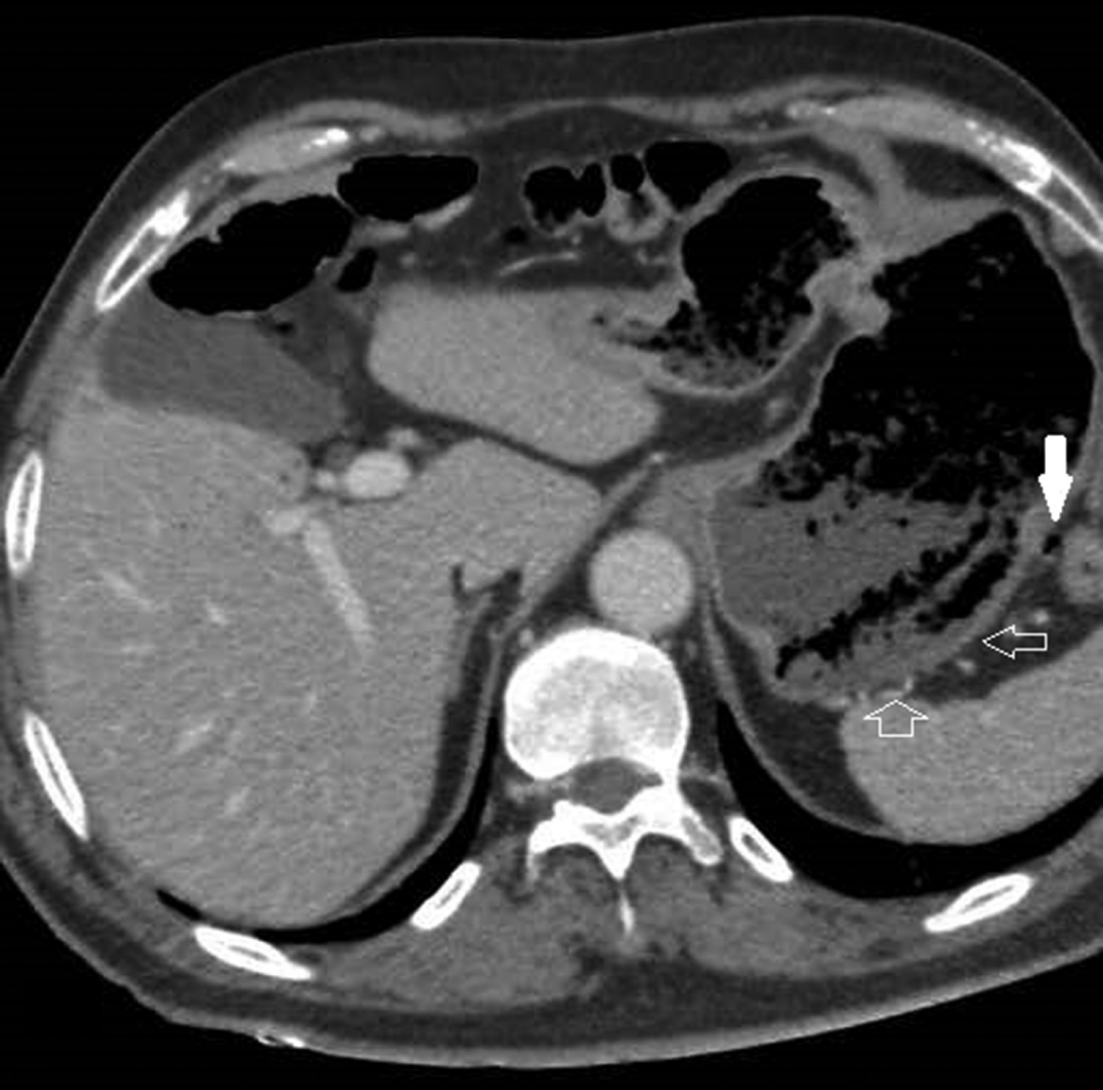
Contrast-enhanced abdominal CT scan in the portal phase. Hypoattenuating posterior gastric wall (open arrows). Ectopic air bubbles inside posterior gastric veins (solid arrow).

**Figure 2.**
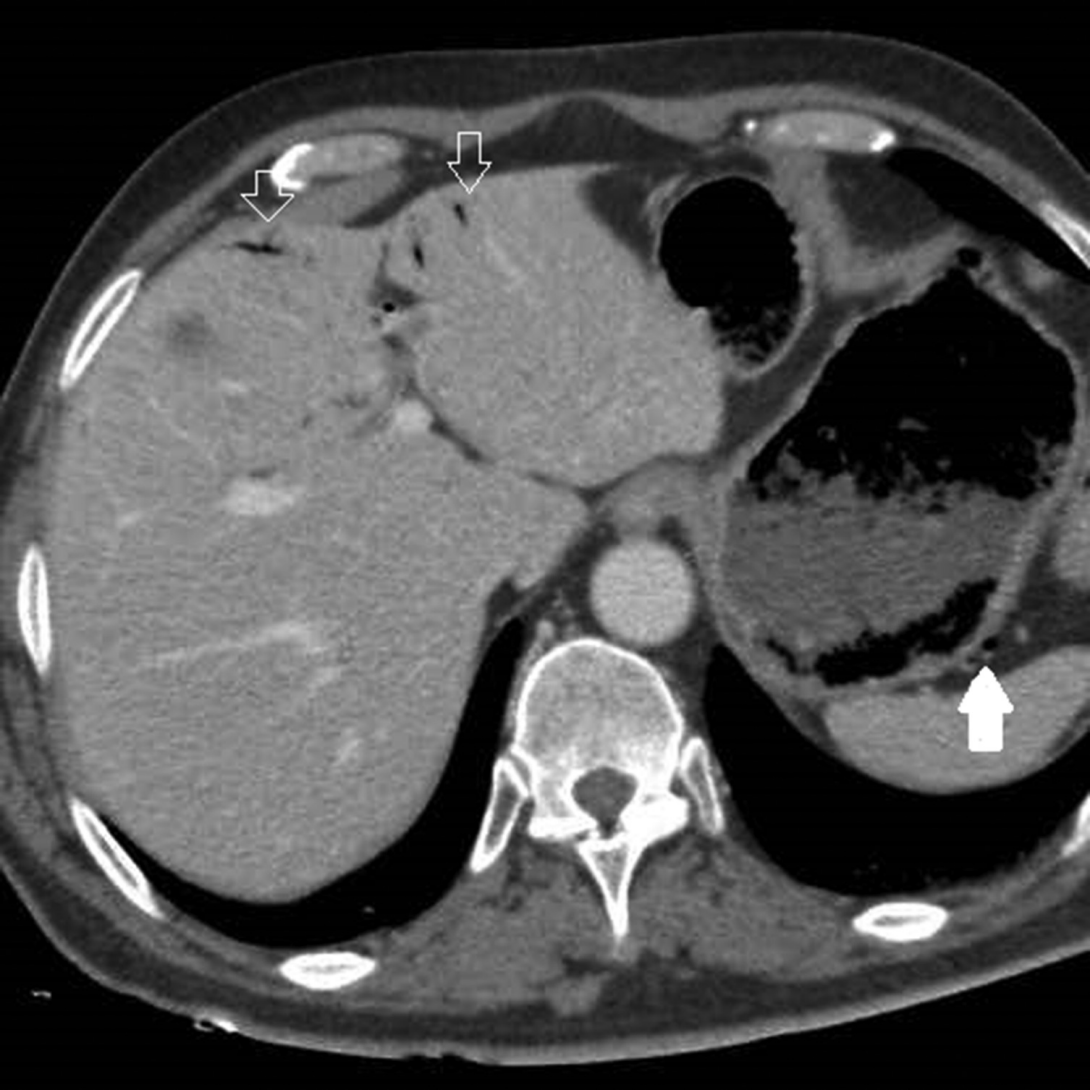
Same scan as in Figure 1 showing ectopic air bubbles in the posterior gastric (solid arrow) and intrahepatic portal veins (open arrows).

**Figure 3.**
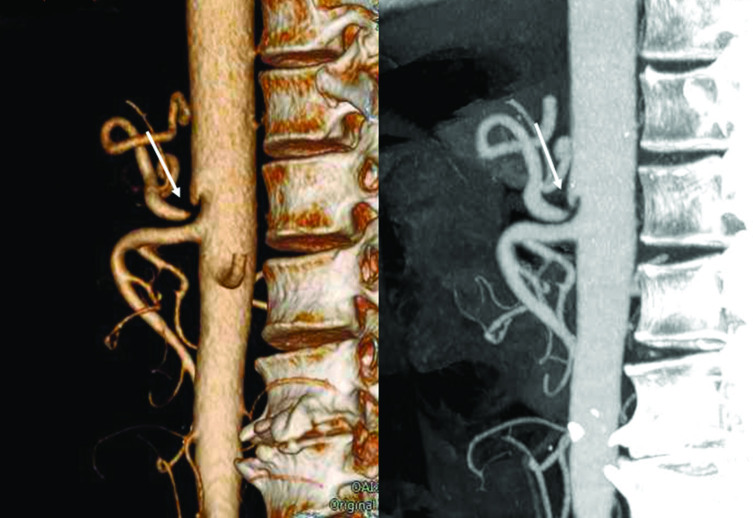
Three-dimensional and maximum intensity projection reformatted images of the CT angiography showing a complete stenosis of the coeliac axis origin with an extrinsic hook-shaped compression (arrows).

**Figure 4.**
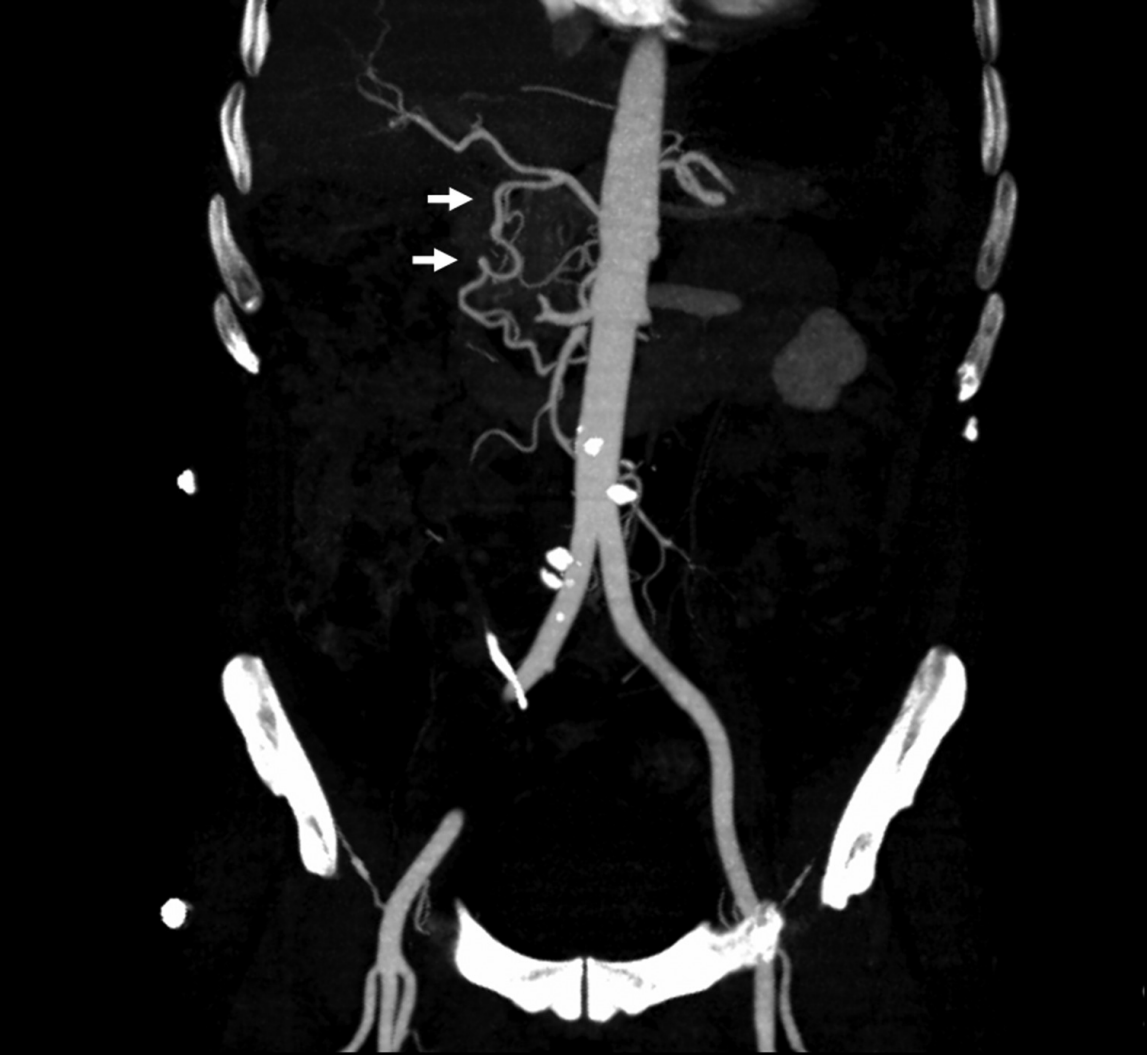
Coronal reformatted image of CT angiography demonstrating (arrows) the hypertrophied gastroduodenal artery.

An abdominal multidetector CT scan was performed in order to rule out abdominal bleeding. The standard protocol in this situation includes a non-enhanced scan that is followed by scans in the arterial and portal phases after the injection of intravenous contrast. Selective volume rendering reconstructions of the abdominal arterial system were carried out. Gastric emphysema was present, exclusively affecting part of the posterior gastric wall, which also showed marked hypoattenuation (Figure 1). Ectopic air was also present, filling the adjacent gastric veins and in the intrahepatic portal vein territory (Figures 1 and 2). The images obtained also demonstrated a complete compression of the coeliac axis origin with a hook-shaped contour and absence of atherosclerotic changes (Figure 3). A compensatory hypertrophied gastroduodenal artery was present (Figure 4). The right and left gastric arteries were normal, with no aberrant course and not showing anatomical variations. No signs of gastrointestinal bleeding were observed. The rest of the abdomen was normal.

An urgent gastroscopy was performed owing to the imaging findings reported. Diffuse erosions with intense mucosal discolouration and inflammation were described, affecting the posterior wall of the fundus and upper gastric body. An incidental antral peptic ulcer was also found in the prepyloric region. No active bleeding was present. Images of the gastroscopy are not available.

## Differential diagnosis

Gastric origin causes of gastric emphysema that should be considered include: emphysematous gastritis, ischaemia, caustic ingestion, increased intragastric pressure from gastric outlet obstruction, gastric mucosal disruption, and traumatic and idiopathic causes. Extragastric causes to consider are air dissection from the mediastinum, and small or large bowel ischaemia. In the absence of fever or other signs of infection emphysematous gastritis was ruled out in our patient. Chest was partially included in the scan. The CT scan did not show alterations in the rest of the abdominal and chest organs. No interventional procedures had been previously performed on the patient, which ruled out an instrumentation-related injury. No caustic ingestion was reported by the patient or his relatives and the clinical presentation was not consistent with this option.

The combination of radiological findings (focal gastric wall pneumatosis, characteristic hooked configuration of the stenosis and hypertrophy of the gastroduodenal artery) and the clinical manifestations left the option of a gastric ischaemia in a median arcuate ligament compression syndrome context as the most probable explanation for the events.

## Treatment

No surgical or endovascular interventional procedures were performed; only a nasogastric tube was placed. Conservative management included volume restoration, intravenous proton pump inhibitor therapy, broad-spectrum antibiotic therapy and blood transfusion.

## Outcome and follow-up

**Figure 5.**
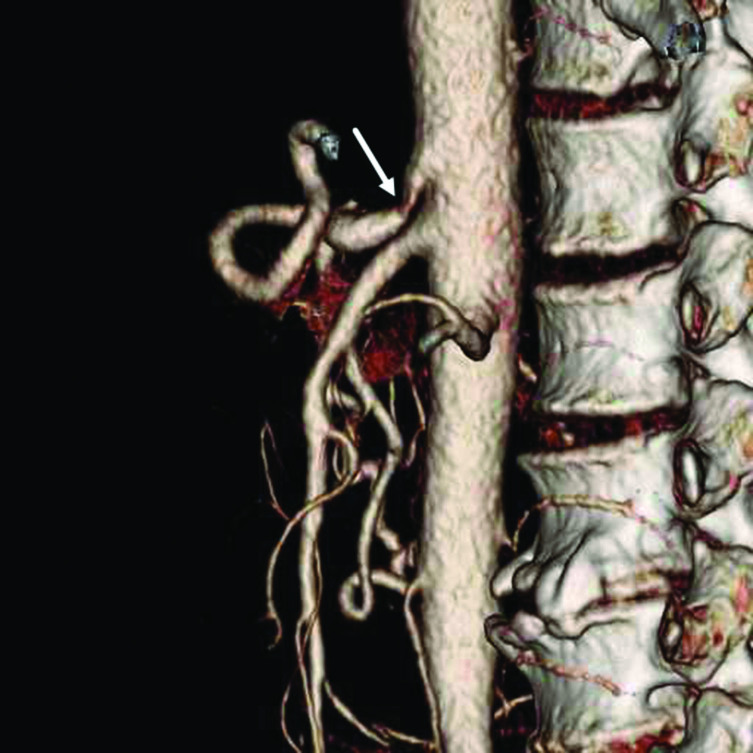
Sagittal CT three-dimensional angiography performed after treatment showing minimum recanalization of the coeliac axis origin (arrow).

**Figure 6.**
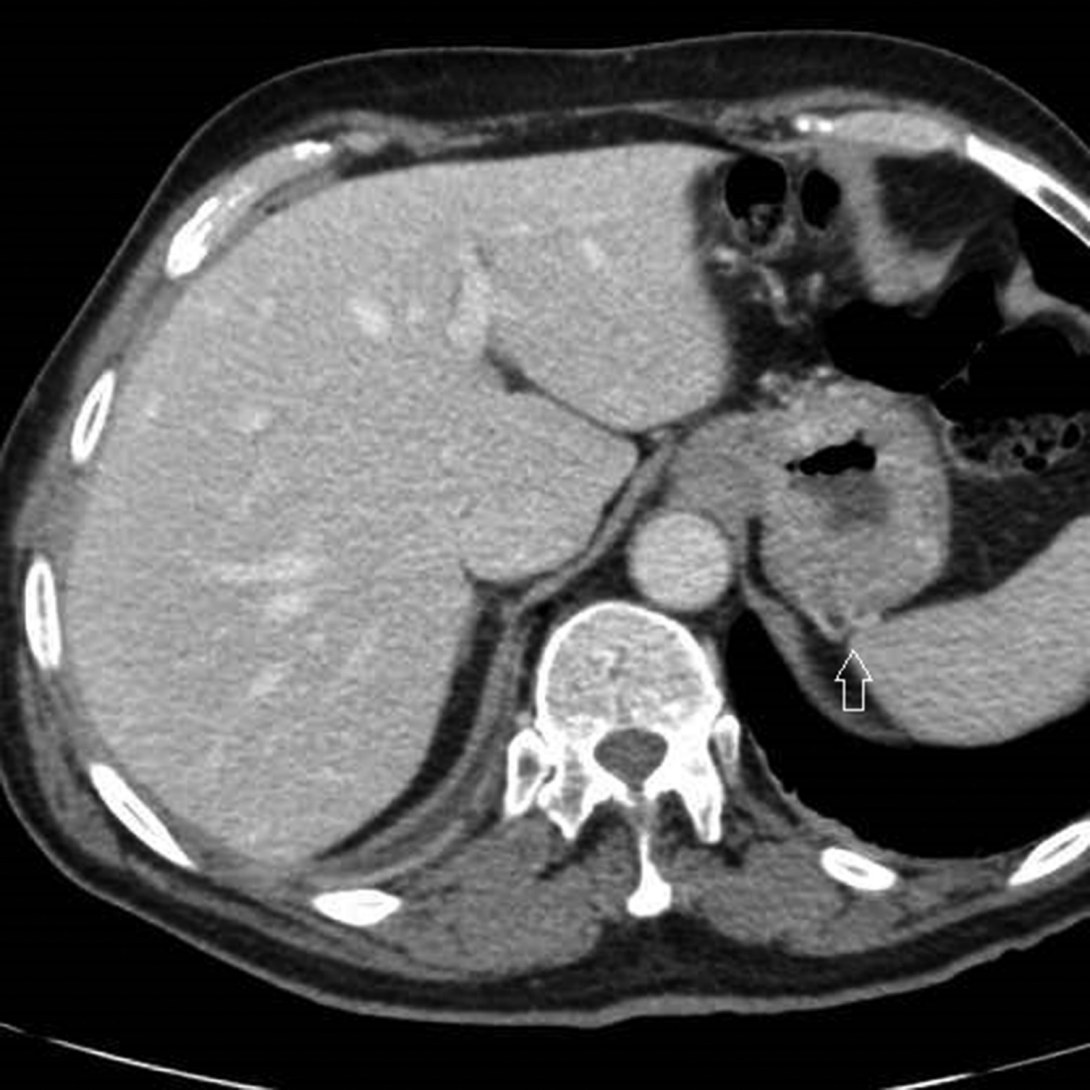
Contrast-enhanced abdominal CT scan in the portal phase performed after treatment showing complete recovery of the posterior gastric wall (arrow) and disappearance of the ectopic air bubbles in the gastric veins and intrahepatic portal territory.

Symptoms gradually subsided with conservative management. A CT scan performed 2 weeks after the onset of the disease showed minimum recanalization of the coeliac axis (Figure 5) and complete disappearance of the ectopic air (Figure 6). Complete clinical recovery was observed after 3 weeks. The patient was discharged and treatment for the incidental antral ulcer was prescribed. Endoscopy performed 3 months after the episode (images not available) showed scarring of the antral ulcer and complete recovery of the rest of the gastric mucosa. After 7 months, the haemoglobin level remained normal and the patient remained asymptomatic.

## Discussion

The median arcuate ligament is a fibrous arch that passes superior to the origin of the coeliac axis and unites the diaphragmatic crura on either side of the aortic hiatus. The first anatomic description was made in 1917.^1^

The median arcuate ligament compression syndrome is an anatomic and clinical entity defined by a combination of extrinsic compression of the coeliac axis by the median arcuate ligament and epigastric pain, nausea, vomiting and weight loss. Patients are usually women with thin body habitus. The pain worsens after meals and knee–chest position may provide relief. Fear of food may also be present.

A higher origin of the coeliac axis or an exuberant growth of neurofibrous tissue originating from the coeliac plexus are some of the theories given to explain the pathogenesis of this condition.^2,3^ The symptoms derived could be explained by postprandial blood theft and an overstimulation of the coeliac plexus, resulting in vasoconstriction and ischaemia.^4,5^

Lateral aortic angiography is traditionally the gold standard but CT angiography with three-dimensional imaging provides a complete and non-invasive evaluation.^6^

CT angiograms demonstrate a typical hook-shaped indentation of the superior aspect of the coeliac axis origin.^7^ The chronic compression of the coeliac axis produces histological changes in the coeliac axis wall, including intimal hyperplasia, proliferation of elastic fibres and disorganization of the adventitia, which sometimes explain the failure of the median arcuate ligament reparation.^8^

The first description of the syndrome *in vivo* was made by Harjola^2^ in 1963 and the first series of treated cases was published by Dunbar et al^9^ in 1965. According to Lindner et al,^10^ in 10%–24% of cases, the ligament may cross anterior to the coeliac axis. Bron et al^11^ in 1969 and Colapinto et al^12^ in 1972 found radiological signs of compression of the coeliac axis in 12.5% and 31% of asymptomatic patients, respectively. Up to 13%–50% of healthy patients during expiration show angiographic features of compression and the majority experience no symptoms.^11^ Levin and Baltaxe^13^ reported 50 asymptomatic patients with coeliac stenosis.

Existence of median arcuate ligament syndrome has been debated because of the high frequency of asymptomatic patients with coeliac axis compression. This may be explained by the rich collateral circulation existing in the splanchnic vasculature, which prevents ischaemia in the presence of single-vessel disease.^14,15^ Current medical literature on gastric ischaemia is limited and generally indicates that this condition is extremely rare because of rich gastric vascular supply.^16^

Ischaemic gastric ulcerations are described to occur often at gastric sites unusual for a peptic ulcer and near the anastomoses between the two arterial arches from the lesser to greater curvature, along the anterior and posterior gastric walls. The diagnostic work-up of gastric ischaemia includes endoscopy and imaging studies.^16^ The most common symptom is severe gastrointestinal bleeding; pain was only rarely noted by the patients.^17^

A CT scan identifies the presence of pneumatosis and wall changes as thickening or thinning and hyper- or hypoattenuation, depending on the type of ischaemia.^18^ It also helps to rule out other intra-abdominal causes and assess for the presence of predisposing conditions.^19^

Our patient presented with severe stenosis of the coeliac axis with the characteristic features described and a significant compensatory hypertrophy of the gastroduodenal artery. The patient remained asymptomatic owing to collateral circulation with probable reversed flow in the gastroduodenal artery (Figure 4). No evidence of haemorrhage was detected. The anaemia observed was probably chronic and caused by the antral peptic ulcer. A low output situation with intense hypotension was triggered by the diarrhoea and dehydration. The posterior gastric wall receives arterial flow from two different vascular inputs: the left gastric artery and the short gastric arteries arising from the splenic artery. This condition creates a virtual watershed area between them that receives blood flow from the most distal branches of each territory. Chronic anaemia and sudden hypotension compromised the blood supply of a poorly irrigated coeliac axis territory that was mainly supplied collaterally by the gastroduodenal artery (Figure 4). Ischaemia occurred in probably the most vulnerable vascular area of the patient's gastric wall. The ischaemic injury caused the intense epigastric pain and produced air bubbles in the posterior gastric wall, and their translation to the mesenteric and portal vein territories. The supporting measures, including volume restoration and blood transfusion, conditioned elevation of the blood pressure with consequent restoration of gastric blood irrigation and recovery of the gastric wall. Clinical and imaging improvement occurred even with minimum recanalization of the coeliac axis (Figure 5). This recanalization was probably secondary to blood pressure normalization, especially because there were no differences in the respiratory phases between the initial and follow-up CT scans that could explain the finding. The ectopic air in the intrahepatic portal system disappeared completely, which supports its association with a non-life-threatening and, therefore, not being always an ominous sign^20^ (Figure 6).

The medical management of gastric ischaemia includes fluid resuscitation, nasogastric tube placement, aggressive acid reduction therapy with intravenous proton pump inhibitors, and selective use of broad-spectrum antibiotics if sepsis or gastric pneumatosis is present. Surgery, instead of supportive care and broad-spectrum antibiotic therapy, is indicated for gastric perforation and non-responding gangrenous or necrotizing gastritis.^16^ Surgical treatment comprises the section of the ligament, carried out by an open or laparoscopic approach. If persistent stenosis is observed, angioplasty, bypass or endovascular treatments are proposed.^4,21^ No interventional procedure was performed on this patient because of the great response to conservative measures.

## Learning points

1. Median arcuate ligament syndrome is an anatomic and clinical entity. Both clinical and radiological findings are needed to establish this diagnosis. A compression of the coeliac axis is frequently present, which causes symptoms in only a few individuals.
2. Severe complications may develop in certain situation owing to failure of compensatory mechanisms. Collateral flow should always be evaluated in order to understand the physiopathology of the syndrome.
3. The characteristic hook-shaped stenosis of the coeliac axis origin appearing on CT angiography helps to rule out an atherosclerotic cause for the disease and prompts the diagnosis. CT angiography provides an excellent and complete evaluation of the median arcuate ligament syndrome, including its complications.
4. Portal pneumatosis observed on CT scan is not always an ominous sign.
5. In gastric ischaemia, conservative management should be considered before other aggressive procedures, especially in emergency situations.

## Consent

Written informed consent from the patient was obtained. This includes use of data, case history and figures. The figures are sufficiently anonymized.
